# Comminuted Fracture of the Skull, Followed by Metastatic Pneumonia and Gangrene

**Published:** 1876-10

**Authors:** J. W. Burns

**Affiliations:** Lake Crystal, Minn.


### EXAMINER.

Vol. XXXIII.—OCTOBER, 1876.—No. 10.

### Original Communications.

### COMMINUTED FRACTURE OF THE SKULL, FOLLOWED BY METASTATIC PNEUMONIA AND GANGRENE.

By J. W. BURNS, M.D., Lake Crystal, Minn.

Thomas M., a tramp, aged about sixty years, was struck on the evening of April 26, 1876, with a heavy club on the left temporal region, and remained out doors during the entire night, which was quite cold. The following morning, when first seen, he presented the following symptoms: The scalp over the left temporal fossa was badly contused and ecchymosed, and a great degree of swelling had taken place. The swelling and ecchymosis extended to both orbital regions, and the eyes were both closed, so that I found it impossible to open them. The countenance was of a deadly pallor; respirations feeble and noiseless; pulse weak, intermittent, and somewhat frequent; pupils contracted, but responded to light; deglutition performed with difficulty; mind in a state of abeyance, but confused; the patient answering questions with great difficulty, and only in monosyllables; bladder and bowels acted normally, paralysis was not manifest, although the voluntary muscles were much weakened. The case was diagnosed as concussion of the brain, with fracture and depression of the skull.

The patient received the usual treatment adopted in concussion of the brain, but nothing like full reaction occurred; still the case gradually improved up to the end of the first week.

May 4th. Discovered a slight paralysis of the right side, and about the same time the pulse became fuller and slower, the pupils more dilated, the breathing labored and slower; deglutition performed with greater difficulty, and the urine and feces were passed involuntarily.

These symptoms gradually increased in severity until the 7th and 8th, when severe convulsions occurred. On the appearance of these the patient was given active mercurial cathartics, and put upon 5 grs. of iodide of potassium and 30 drops of fl. ex. ergot, four times a day. The case now began to improve, and in the course of a week the paralysis had entirely disappeared and the bodily functions were performed naturally; the mind grew stronger, but the pulse continued slow and full; the respirations slow but performed easily; the pupils were normal. No inflammatory action has been manifest in the case.

May 24th. The patient is able to walk a mile or two every day, has a good appetite, and is improving mentally and physically; the pulse and respirations are preternaturally slow, the mind weak and somewhat confused by spells.

At no time during the treatment had the patient complained of severe pain in the head; he occasionally said that it was "sore." At the seat of injury a fracture could be distinctly traced, below the temporal ridge, extending backwards and upwards through the parietal bone towards the sagittal suture. The depression in the temporal fossa was well marked, but as the patient presented no symptoms that warranted surgical interference, he was discharged and left the village about June 1st. I cannot obtain a history of the case from the 1st to the 16th inst.

On the latter date he was found twenty miles from here and taken to Mankato, where he was attended by the county physician, who found him in an insensible condition, and dying from some disease of the lungs. Death occurred on the 20th inst.

From the attending physician I learn that he remained in a semi-comatose condition up to the time of death; that the pulse and respirations were greatly accelerated; that urine and feces were passed involuntarily, and that he expectorated large quantities of a brownish fetid material.

A *post mortem* examination of the head, (at which I was not present,) was made on the day of death, and revealed a comminuted fracture in the left temporal fossa, involving the great wing of the sphenoid, a portion of the frontal, the squamous portion of the temporal and the parietal below the temporal ridge. The fracture covered a space $1\frac{1}{2}$ by $2\frac{1}{4}$ inches. Besides this there was a fracture five inches in length commencing in the frontal bone below the temporal ridge, and extending backwards and upwards to the sagittal suture. The greatest depression was $\frac{5}{16}$ of an inch, and occurred in the parietal bone below the temporal ridge. The under surface of the fracture was comparatively smooth, except at one or two points which had evidently punctured the membranes and caused an effusion of serum.

A slight sanguineous effusion had taken place between the dura mater and skull, and also in the substance of the brain, which was slightly softened in the immediate vicinity of the greatest depression. No effusion had occurred in the ventricles, and the remaining portion of the brain appeared normal. Examination of the thorax revealed three bluish-green moist sloughs in the parenchyma of the upper lobe of the right lung. They varied in size from a hickory-nut to a hen's egg, and were not abruptly limited, but merged into the hepatized parenchyma. They contained a greenish-black stinking substance, saturated with blackish-gray ichor.

Mortification had reached the pleura at one point, and its surface was bathed with this same greenish-black substance. Extensive adhesions were present in the upper part of the pleura. A bronchus had opened into one of the sloughs, hence the copious-brownish expectoration previous to death.

The remaining slough was circumscribed. At one point in the middle lobe, a dark, yellowish deposit had occurred on the pleura, and its surfaces were somewhat adherent, and beneath was a rounded nodular prominence. A similar prominence was found in the left lung, just beneath the pleura, but with less deposits. The remaining portion of the right lung was bathed in a reddish-gray matter, and it could be expressed in large quantities from its cut surface, which was of a grayish-yellow color. The tissues were tender and easily torn; a slight granular appearance was visible. The left lung was in a hyperemic condition. This, with the condition described above, were the only changes noticed. The heart and abdominal viscera were all in a normal condition.

A point of considerable interest in the case was the amount of depression without symptoms of compression during the first week. I attribute the paralysis and other symptoms of compression that occurred a week or ten days after the injury to effusion of serum from puncture of the membranes, and the sanguineous effusion in the substance of the brain, and not to direct compression by the bone.

Another point of interest is the lung difficulty. Was the pneumonia metastatic, or did it originate from exposure and the debilitated condition of the patient? The evidence I think is in favor of the former. Abscesses and gangrene are very rare as a sequel of pneumonia, even in old and debilitated cases, while hæmorrhagic infarction following injuries of the skull where the diploe has been penetrated is of a frequent occurrence, and is due to the gaping of the walls of the veins, which being adherent to the tables of the skull, are prevented from closing, so that entrance of coagula into them is facilitated.

Hæmorrhagic infarction had certainly taken place at two points, as shown by the dark yellowish deposit on the surface of the pleura, adhesion of its surfaces and the rounded, nodular prominence immediately beneath. That the gangrenous abscesses or sloughs were at first simple infarctions I have no doubt, but owing to extravasation and compression of the capillaries, secondary coagula formed in the bronchial arteries and nutritive vessels, thus withholding nutritive material from the infarction and causing its death.

The principle upon which metastatic infarctions occur caused by emboli from sanious or suppurating surfaces is too well understood by the profession to call for any remarks.

That such infarction occurred in this case, and that abscess, grangrene and pneumonia followed is, I think, well established by the history and *post mortem*. The patient evidently died from metastatic disease of the lungs, induced or caused by the injury of the skull and brain.

I will not open the question whether an operation was advisable or not during the time I treated the case, but leave it for each surgeon to decide for himself.

